# Measuring Visual Field Progression in the Central 10 Degrees Using Additional Information from Central 24 Degrees Visual Fields and ‘Lasso Regression’

**DOI:** 10.1371/journal.pone.0072199

**Published:** 2013-08-12

**Authors:** Ryo Asaoka

**Affiliations:** Department of Ophthalmology, University of Tokyo Graduate School of Medicine, Tokyo, Japan; Saitama Medical University, Japan

## Abstract

**Purpose:**

To measure progression of the visual field (VF) mean deviation (MD) index in longitudinal 10-2 VFs more accurately, by adding information from 24-2 VFs using Lasso regression.

**Methods:**

A training dataset consisted of 138 eyes from 97 patients with glaucoma or ocular hypertension and a testing dataset consisted of 40 eyes from 34 patients with glaucoma or ocular hypertension. The Lasso method was used to predict total deviation (TD) values in training patients’ 10-2 VFs based on information from their 24-2 VFs (52 TD values, foveal sensitivity and mean deviation MD). Then, the MD of each patient’s 10-2 VF was estimated as the average of these Lasso-predicted TD values (10-2 VF ‘Lasso MD’; LMD). Finally, linear regression was applied to each testing patient’s series of longitudinal 10-2 VF MDs with and without additional Lasso-derived LMDs in order to predict future MDs not included in the regression analysis. Absolute prediction errors were compared when only actual 10-2 MDs were regressed against when a combination of actual 10-2 MDs and LMDs were regressed.

**Results:**

The average absolute prediction error was significantly smaller for the novel method incorporating LMDs (range: 1.6 to 1.8 dB) compared with the standard approach (range: 1.7 to 3.4 dB) (p<0.05, ANOVA test).

**Conclusions:**

Deriving 10-2 VF MD values from 24-2 VFs improves the prediction accuracy of progression. This approach will help clinicians to predict patients’ visual function in the parafoveal area.

## Introduction

Glaucoma is one of the leading causes of blindness in the world [1], [2]. Glaucomatous visual field (VF) change usually manifests in the mid-peripheral VF, while the central region tends to retain visual function until late on in the disease process. In advanced glaucoma, VF damage is often characterized by large arcuate scotomata in the upper and lower hemifields, which have connected to form a ring, threatening visual function in the central area of the VF [3], [4]. Paracentral VF defects are especially important because VF damage in this area leads to disability in various daily tasks [5], and increases the risk of falls, hip fractures and mortality [6], [7]. Thus treatments should be intensified when the rate of VF damage threatens the patient’s visual function, particularly in the central region. In consequence, predicting glaucomatous VF progression in the central area is one of the most important tasks faced by a clinician. Moreover, parafoveal defects occur primarily in early glaucoma [8]–[11], probably due to a distinctive pathological mechanism [8]. It has been suggested that patients with paracentral defects are best monitored by VF testing using a central 10° program [12], [13]. However, one of the hurdles in obtaining a measurement of the central 10° VF is the need for an extra-measurement, in addition to the usual 24 degrees or 30 degrees VF test pattern. Hence we often omit one or other of the two measurements on a given clinic visit. Therefore, measuring 10° VF progression is difficult since test patterns are not consistent, and the number of 10° VF is often small.

One of the most frequently used methods to forecast progression in a patient’s VF is to apply ordinary least squares linear regression (OLSR) analysis to a series of mean deviation (MD) measurements. This method is known as a trend analysis and is employed in the Humphrey Field Analyzer (HFA, Carl Zeiss Meditec, Dublin, CA). In this study, we propose a method to predict MD in the 10-2 VF using information gained from 24-2 or 30-2 VF test results. These values can be used as an additional surrogate measure of MD in the 10-2 VF and as a result, this approach can improve the prediction accuracy of future VF change.

## Method

The study was approved by the Research Ethics Committee of the Graduate School of Medicine and Faculty of Medicine at the University of Tokyo. Written consent was given by the patients for their information to be stored in the hospital database and used for research. This study was performed according to the tenets of the Declaration of Helsinki.

This retrospective study included a training dataset of 138 eyes from 97 patients with a diagnosis of primary open-angle glaucoma, normal tension glaucoma, secondary open angle glaucoma, primary angle closure glaucoma or ocular hypertension. In addition, a testing dataset consisted of 40 eyes from 34 patients with a diagnosis of primary open-angle glaucoma, normal tension glaucoma or secondary open angle glaucoma. Patients were followed in the general glaucoma clinic at the University of Tokyo Hospital. Patients who underwent HFA measurements with both the 30-2 or 24-2 VF test pattern *and* the 10-2 VF test pattern, no longer than 6 months apart, were included. Cases where the 10-2 VF was measured at least six times were entered into the testing dataset, otherwise VFs were used in the training dataset.

Other criteria for inclusion in the study were visual acuity better than 6/12, refraction less than 5 dioptre ametropia, no previous ocular surgery (except for cataract extraction and intraocular lens implantation), and no other posterior segment eye disease. All VFs were recorded using the SITA standard strategy with a Goldmann size III target. Reliability criteria applied were fixation losses less than 25% and false-positive responses less than 15%, a false-negative rate was not used to exclude VFs based on results in [14]. Patients who underwent intraocular surgical treatments during the observed period were excluded from the analysis. The VF of right eye was mirror-imaged to that of a left eye.

The rate of progression of MD, calculated as the mean of pointwise TD values, was calculated using OLSR for each training patient’s series of 10-2 VFs, and for the same patient’s series of 24-2/30-2 VFs. When the patient’s VF was measured using the 30-2 program, only the 52 test points overlapping with the 24-2 VF test pattern (excluding points corresponding to the blind spot) were used to calculate MD, following the analysis performed by the Humphrey Guided Progression Analysis™ (GPA) software.

### Novel Linear Regression Analysis: Lasso Regression

VF trend analyses are usually performed using OLSR. Estimates from OLSR often have low bias but large variance. This can be improved by shrinking or setting some coefficients to zero [15]; as a result, bias is sacrificed in order to reduce the variance of the predicted values [15]. Lasso regression is one approach to constrain regression coefficients. The method requires that the sum of the absolute values of the standardized estimated coefficients is less than a constant, λ. Mathematically, this is denoted:
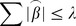
where 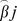 are the estimated coefficients [16].

In this research we applied Lasso regression to VF measurements in order to assess its usefulness for improving prediction accuracy of future VF damage.

### ‘Training’ and ‘Testing’ the Proposed Method

In the training dataset, using 24-2 and 10-2 VFs, OLSR was carried out on all points in the 24-2 VF for its series of TD values as well as the MD value and foveal sensitivity to predict each TD value in the corresponding 10-2 VF; a Lasso λ value was selected which yielded the minimum squared error in a ten-fold cross-validation with patients’ corresponding 10-2 VF TDs. As a result, the Lasso formula consists of selected 24-2 VF parameters with various weights was calculated for each TD value on the 10-2 VF. Next, in the testing dataset, 10-2 VF TD values were predicted from 24-2 VFs, using the derived Lasso formulae. The average of these predicted TD values was then calculated to derive a Lasso MD value denoted as “LMDs”. For comparison, the average of the twelve test points within ten degrees from fixation in the 24-2/30-2 VF was also calculated (denoted, MD_12_). The difference between Lasso-predicted MDs and actual 10-2 VF MDs was investigated using the leave-one-out cross validation method [17]; the statistical significance was calculated using the percentile bootstrap method [18].

Subsequently, in the testing dataset, the initial five 10-2 VF MDs, as well as the LMDs, were regressed in order to forecast the MD of the sixth 10-2 VF using OLSR. In these analyses, the LMDs were not included if 10-2 VF measurements were also carried out on the same dates. The prediction error for the forecast was then calculated as the absolute difference between the predicted MD and the actual MD. This analysis was then iterated, removing 10-2 VF MDs, starting from the fifth towards the initial 10-2 VF, in order to further investigate the usefulness of LMD as a surrogate measure of the 10-2 VF MD. Prediction errors derived from Lasso regression are denoted 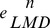 where *n* indicates the number of MDs in the series; for example, 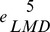 denotes the prediction error for the 6^th^ 10-2 VF MD when using only the first 5 10-2 VF MDs. As a reference, the absolute prediction error was also calculated using OLSR of only actual 10-2 VF MDs; this error is denoted as 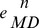. Prediction errors for the reference standard were then compared to the proposed analysis using the one-way ANOVA test.

All statistical analyses were carried out using the statistical programming language R (ver. 2.14.2, The R Foundation for Statistical Computing, Vienna, Austria) and Medcalc version 11.4.2.0; MedCalc statistical software, Mariakerke, Belgium). The R packages “glmnet”, was used to carry out Lasso regression.

## Results

**Figure 1:**
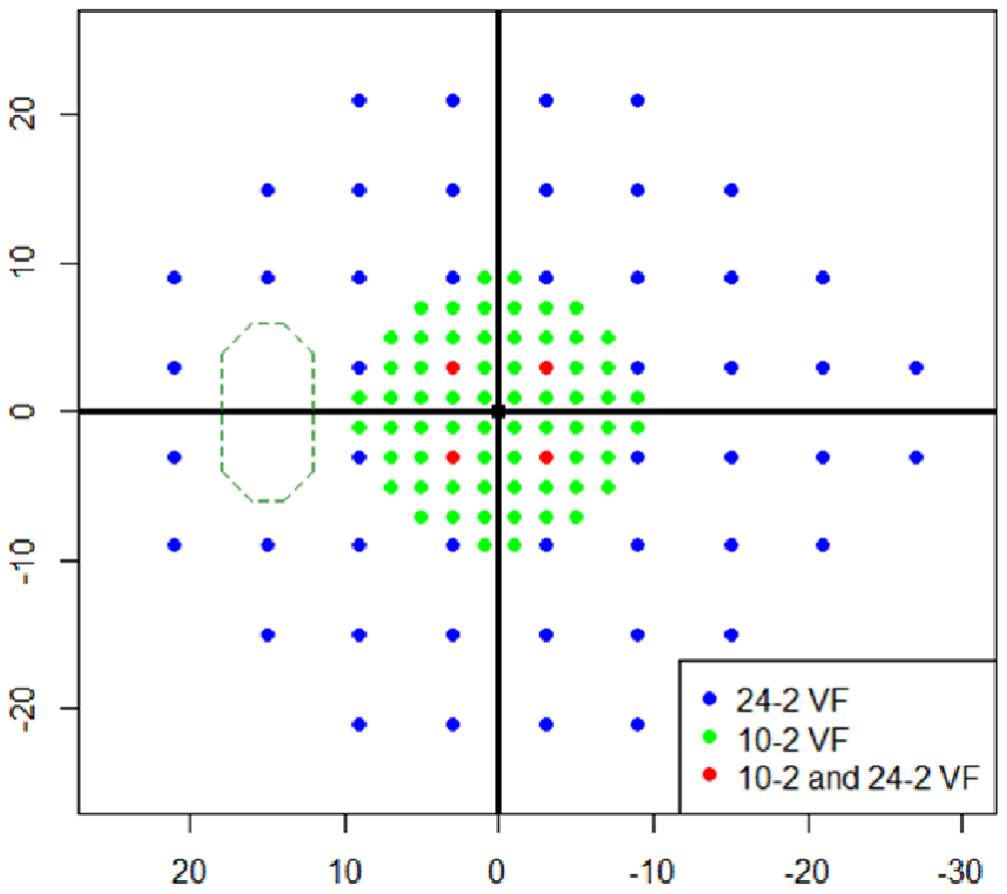
Mapping of 10-2 and 24-2 VF test points. Blue and green circles represent test points in the 24-2 VF and 10-2 VF, respectively. Red circles show points tested by both the 24-2 VF and 10-2 VF. VF: visual field.

Subject characteristics of the training and testing datasets are given in Table 1. The locations of points in the 10-2 VF and 24-2 VF are shown in Figure 1. In the testing dataset, 12.2±3.6 (mean ± standard deviation (SD)) 24-2 VF measurements were carried out over 6.7±1.6 years and six 10-2 VF measurements were carried out over 3.7±1.3 years.

**Table 1:** Characteristics of the study participants of training and testing datasets.

Training		
Age, y, mean ± SD [range]	56.6±12.5 [16.2 to 78.4]	
Gender (Male : Female)	44∶ 53	
Right : Left (eyes)	65∶ 73	
MD of initial 30-2 VF, dB, mean ± SD [range]	−12.8±7.6 [−29.2 to 0.45]	
MD of initial 10-2 VF, dB, mean ± SD [range]	−14.0±8.0 [−30.9 to 0.07]	
Type of glaucoma (POAG, NTG, SOAG, PACG,OH)	52, 75, 8, 1, 2	
Testing		
Age, y, mean ± SD [range]	59.2±12.8 [26.6 to 80.3]	
Gender (Male : Female)	22∶ 12	
Right : Left (eyes)	21∶ 19	
MD of initial 10-2 VF, dB, mean ± SD [range]	−14.2±5.9 [−27.3 to −4.1]	
Type of glaucoma (POAG, NTG, SOAG)	12, 20, 7	

MD: mean of total deviation values, VF: visual field, SD: standard deviation values at the first visit. POAG: primary open angle glaucoma, NTG: normal tension glaucoma, SOAG: secondary open angle glaucoma, PACG: primary angle closure glaucoma, OH: ocular hypertension.

**Figure 2:**
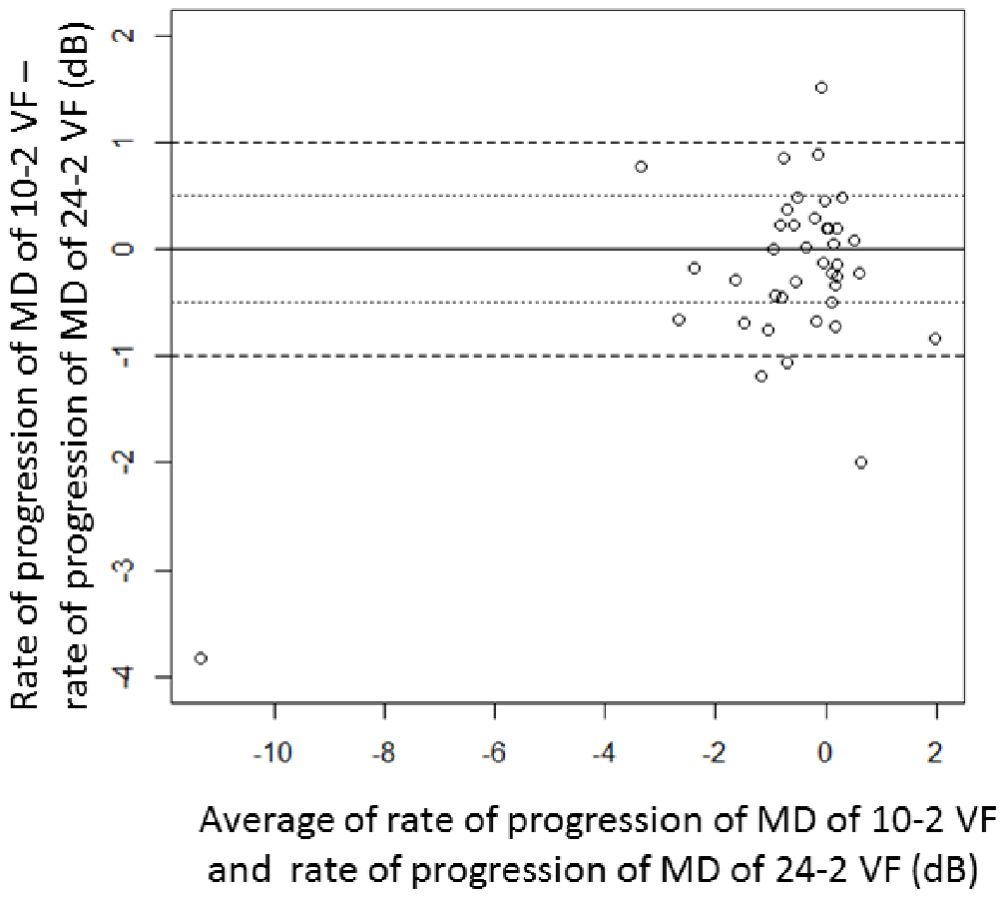
Comparison of rates of progression of MD in the 10-2 VF and 24-2 VF. (Bland-Altman plot). If there was no difference between the pair of measurements, then the values would lie on a horizontal line at zero. In 14 (35.9%) of 39 eyes, the difference of the rates were larger than 0.5 dB/year and furthermore, the difference of the rates were larger than 1 dB/year in four (10.3%) of 39 eyes. MD: mean of total deviation, VF: visual field.

**Figure 3:**
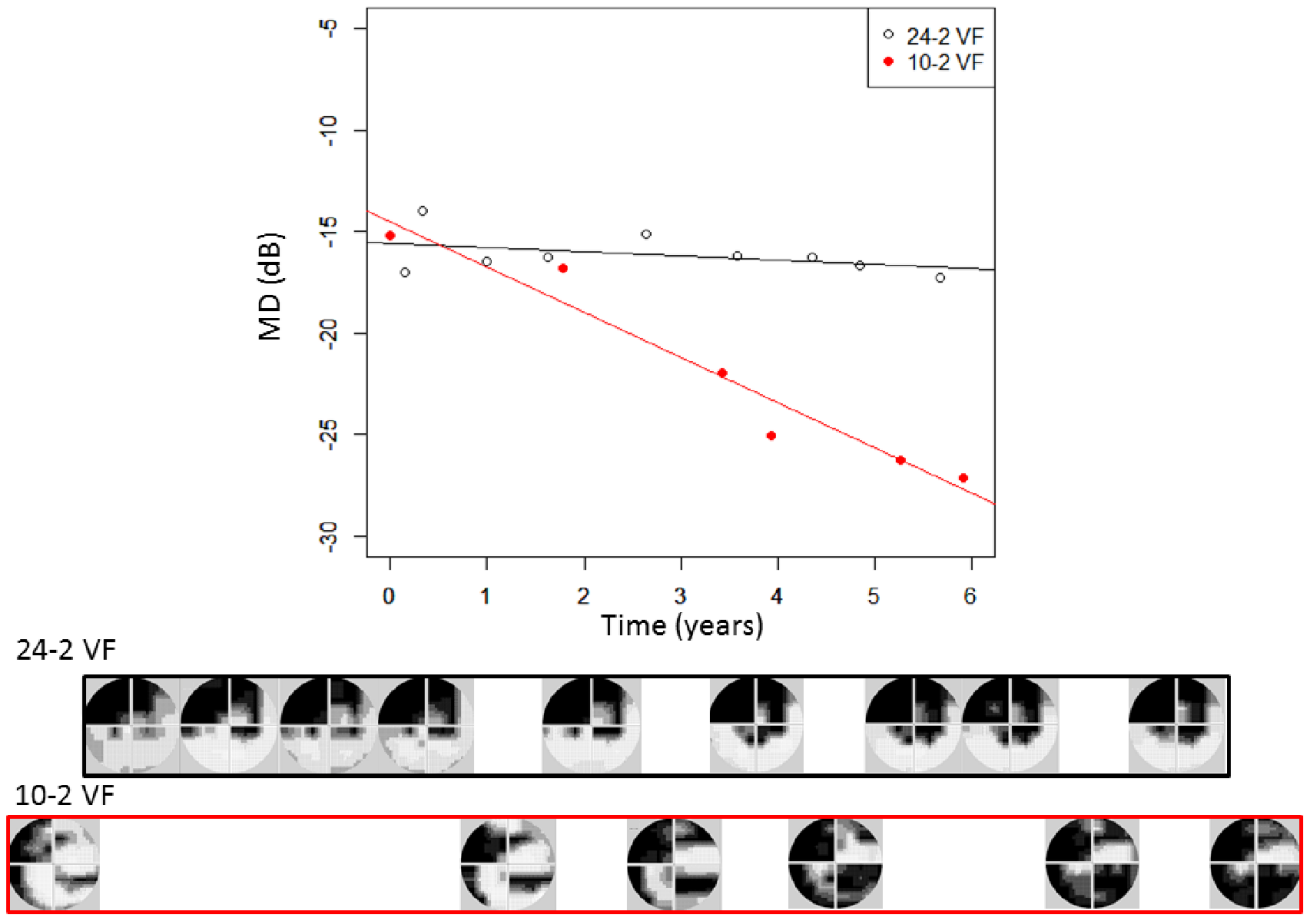
The progression of MD in the 10-2 VF and 24-2 VF in a given patient. In this patient, the rate of progression of MD over the first six 10-2 VFs was −2.3 dB/year whereas over the first nine 24-2 VFs, the MD rate was −0.14 dB/year. MD: mean of total deviation, VF: visual field.

For the testing dataset, the rate of progression of MD in a patient’s series of 10-2 VFs is compared with that in their 24-2/30-2 VFs in Figure 2. One patient is not included in the Figure because he/she underwent only two 24-2/30-2 VFs during the period of receiving six 10-2 VFs. The mean ± SD of the progression rate was −0.8±2.2 dB/year with the 10-2 VF MD and −0.6±1.8 dB/year with the 24-2 VF MD. The mean ± SD difference in rates (absolute value) was 0.58±0.67 [range: 0.002 to 3.8] dB/year. In 14 (35.9%) of the 39 eyes, the difference in rates was larger than 0.5 dB/year and larger than 1 dB/year in four (10.3%) of the 39 eyes. VF progression is illustrated for a single patient in Figure 3; in this patient the rate of progression of MD in their series of 10-2 VFs was −2.3 dB/year, whereas the MD rate was −0.14 dB/year in their 24-2 VF series.

**Figure 4:**
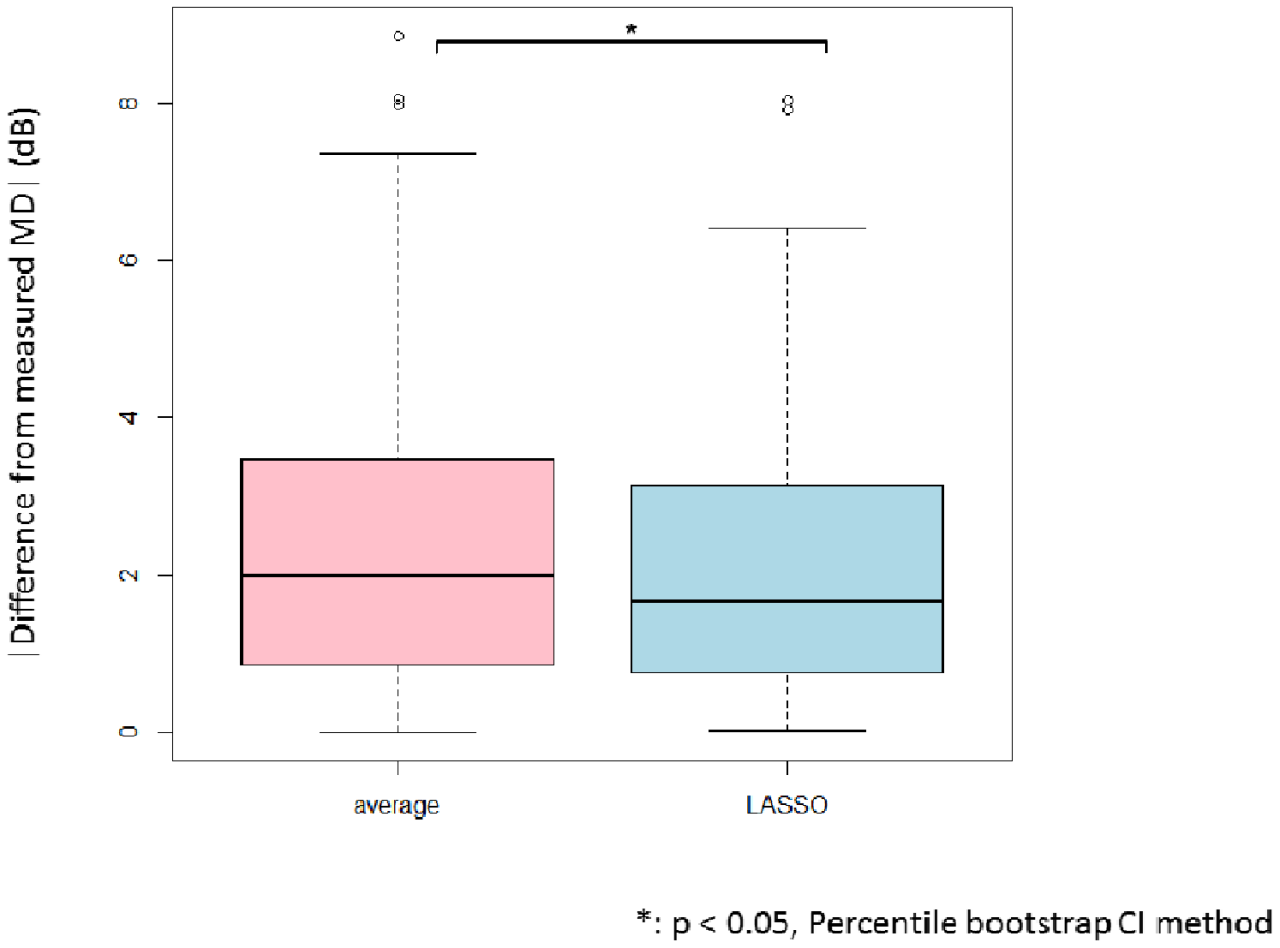
Comparison of the difference of the Lasso-derived MD and the average of the twelve test points within ten degrees from fixation in the 24-2/30-2 VF, against the actual 10-2 VF MD. The Lasso-derived MD was obtained by leave-one-out cross validation. The difference shown is the absolute value. The difference associated with the Lasso derived MD was significantly smaller than that associated with the average of the central twelve 24-2 VF test points (p<0.05, percentile bootstrap method). MD: mean of total deviation, VF: visual field.

The median [first and third quartile] value of the Lasso-derived LMDs and actual measured 10-2 VF MDs was 1.7 [0.8 and 3.1], and that between actual measured 10-2 VF MDs and MD_12_ was 2.0 [0.9 and 3.5] (Figure 4). The difference between actual 10-2 VF MDs andLMDs was significantly smaller than that between actual 10-2 VF MDs and MD_12_ (p<0.05, percentile bootstrap method).

**Figure 5:**
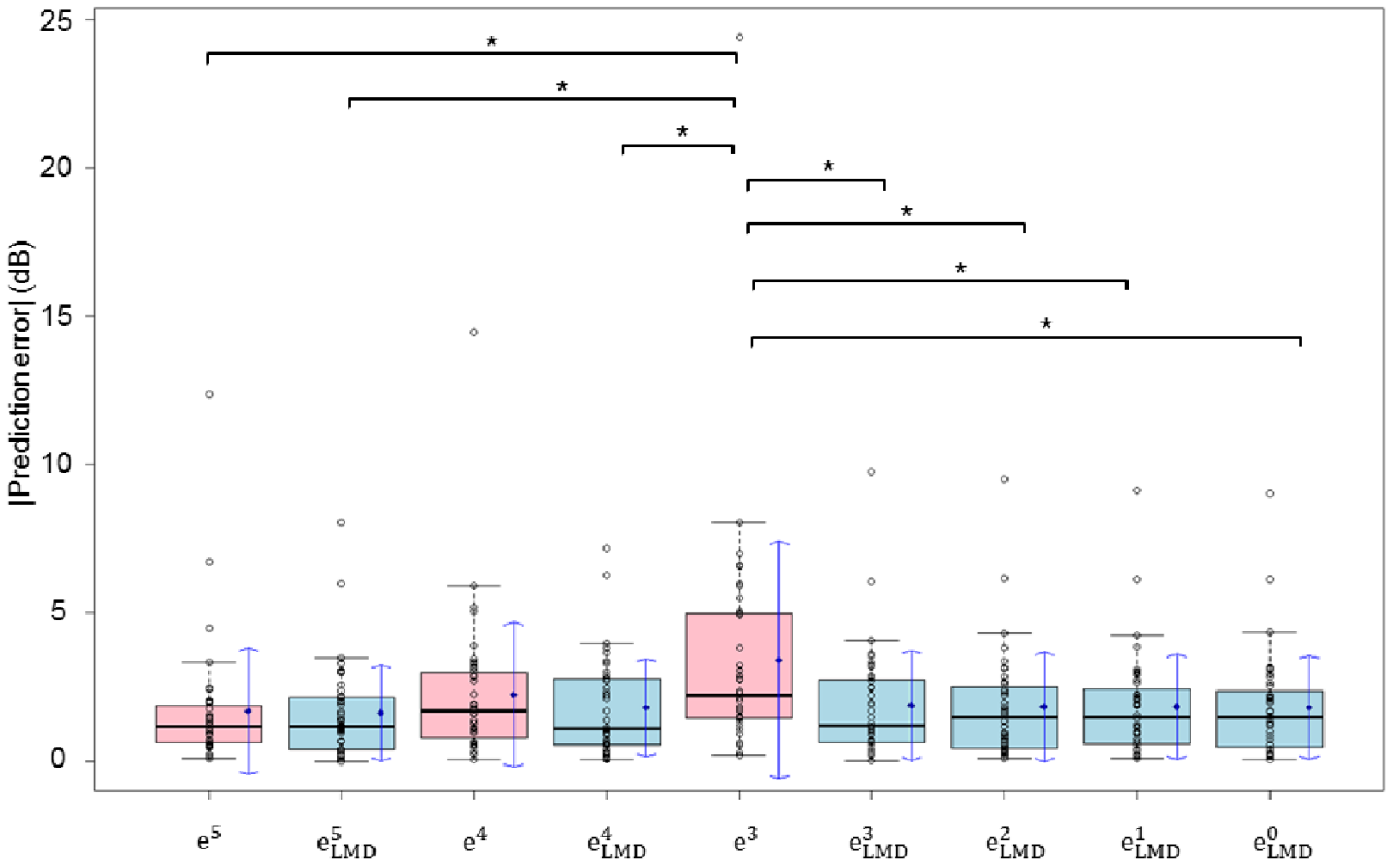
Prediction errors for the novel method are compared with the standard approach. There were significant differences between 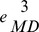 and all other values (p<0.05, one-way ANOVA test). 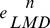 : prediction errors derived from the Lasso regression (n indicates the number of MDs in the series), 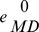: the absolute prediction error calculated using only actual 10-2 VF MDs, MD: mean of total deviation, VF: visual field.

The prediction error associated with regressing only actual 10-2 VF MDs (range: 1.7 to 3.4 dB) was significantly larger than the novel approach (range: 1.6 to 1.8 dB) (p<0.05, ANOVA test); see Figure 5.

**Figure 6:**
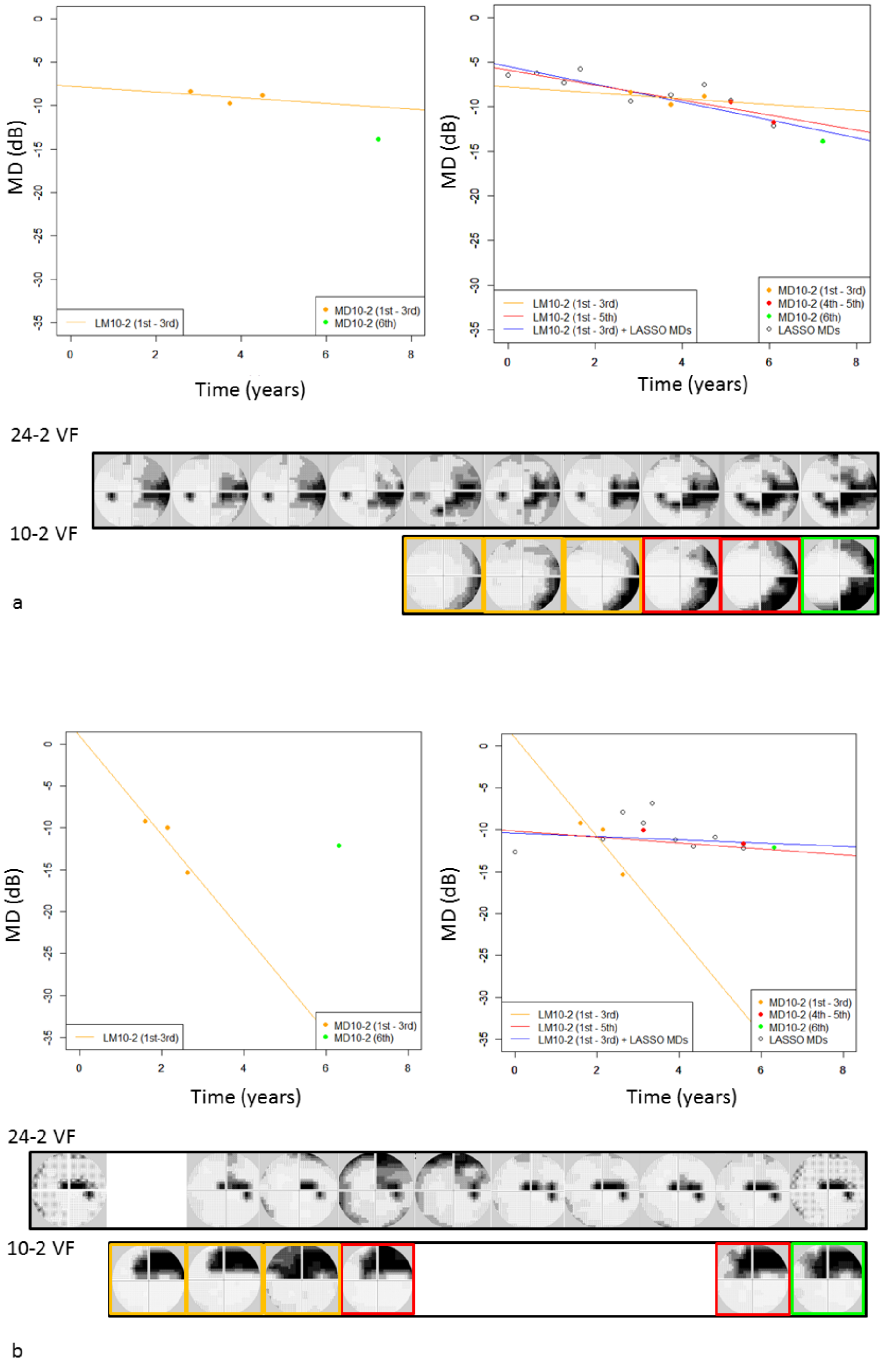
The progression of actual 10-2 VF MDs and LMDs. Orange circles represent three initial actual 10-2 VF MDs, red circles represent 4th and 5th 10-2 VF MDs, the green circle represents the 6th 10-2 VF MD and black circles represent LMDs. Linear regression lines are drawn for the initial five (red; ‘VF10-2 1st to 5th’) and three (orange; ‘VF10-2 1st to 3rd’) actual MDs, and initial three actual MDs plus LMDs (blue; ‘LMD’) in four different patients. The grayscale images illustrate the actual 30-2 VFs and 10-2 VFs. a: case1, 61 years old, male, left eye b: case2, 76 years old, male, right eye. VF: visual field, MD: mean of total deviation, LMD: Lasso-derived 10-2 VF MD.

The progression of MD in the 10-2 VF is illustrated for two sample patients in Figure 6 a and b. Regression lines are colored according to the method applied: OLSR of the initial three 10-2 VFs (orange line), initial five 10-2 VFs (red line), and initial three 10-2 VF MDs plus 24-2 VF derived LMDs (blue line). In this calculation 24-2 VF MD was calculated using 52 test points of 30-2 VF relevant to 24-2 VF.

## Discussion

In the current study, it was observed that the rate of progression of a patient’s MD in their 10-2 VF can be considerably different from that of their 24-2 VF (see Figure 2). We attempted to predict a patient’s 10-2 VF MD by applying the Lasso regression to their 24-2 VF results. Furthermore, we have shown that combining LMDs with actual 10-2 VF MDs resulted in a considerably smaller prediction error when forecasting future change in the 10-2 VF. Thus, a more accurate estimate of central VF progression can be obtained, even when the number of 10-2 VFs is low, by supplementing the ‘gap’ with information from 24-2 VFs.

Few studies have been motivated to investigate the discrepancy between the progression of glaucoma in the 24-2 VF and the 10-2 VF. Suzuki et al. divided the VF into 15 sectors and found that the average TD in each sector correlated with the global MD value to differing magnitudes according to the sector location [19]. Our findings suggest that not only does the cross-sectional relationship of VF sensitivity vary according to the area of the VF, but also the longitudinal relationship (the rates of progression).

Very recently, Su et al. investigated the mode of progression of test points in the 10-2 VF over time, and suggested that the pattern varies according to the location, even within the parafoveal area [20]. Thus, the four test points in the 24-2/30-2 VF that overlap with the 10-2 VF would not be sufficient to evaluate the central VF. On the other hand, VF measurements are imprecise (large variability), and Chauhan et al. reported that three 24-2 VF examinations per year (for 2 years) are required to identify a change in MD of −2 dB/year [21]. If three 10-2 VFs a year should also be carried out to analyze progression in the parafoveal region, in addition to three 24-2/30-2 VFs, six VFs are required a year. This equates to a VF test every two months, or two every four months, which is most likely beyond the capacity of a busy clinic. Indeed the recommended testing interval for VFs by the National Institute for Health and Clinical Excellence (NICE) Guidelines [22] is merely every six to 12 months, unless progression is suspected. The method proposed in this research can be used to predict 10-2 VF MDs from 24-2 VFs, thus ‘filling the gaps’ where 10-2 VF measurements cannot be taken. This can help clinicians to estimate both parafoveal and peripheral progression avoiding additional financial resources, and a burden on physician loads.

A possible caveat of the current study is the relatively small number of 10-2 VFs in each patient’s series (6 VFs). Indeed it has been reported that five [23], eight or an even higher number [24], [25] of VFs is necessary to carry out point-wise linear regression, mainly because of the large noise associated with the measurement. However, as the variability decreases in the central area [22] and also MD is more robust than point-wise sensitivity for noise, investigating only six 10-2 VFs is clinically relevant. Moreover, a recent multi-centre cross-sectional study in England revealed most glaucomatous patients receive less than three VFs in the first 2 years following diagnosis and the average number of VFs undertaken by patients was merely 0.7 per year over the duration of follow-up [26].

The proposed method may also be helpful to reduce the length of observation period required to detect progression. Jansonius et al. have proposed that estimates of speed of loss, and predictions based on that speed of loss, should not be made until a patient has been monitored for more than 5 years [27]. Adding LMDs to the trend analysis, as suggested in the current study was shown to reduce the prediction error, and should help to make accurate estimates of the rate of progression earlier than standard methods allow.

OLSR is a frequentist approach and assumes there are sufficient measurements to extract meaningful information. As VFs are ‘noisy’ measurements with large fluctuations or measurement error, outliers are quite common. Russell et al. propose that estimates of VF progression can be improved by considering structural information to reduce the level of noise [28]. The advantage of the current approach is that it makes use of existing VF data as much as possible and the influence of VF variability is reduced. As a result, it was possible to more accurately evaluate the progression of MD in 10-2 VFs; furthermore, the associated prediction error was not significantly different from using four/five actual 10-2 VF measurements. One of the possible caveat is that the current approach contributed to improve the prediction accuracy compared with the prediction with actual three 10-2 VFs, but not with four/five 10-2 VFs. A future study should be carried out to further improve the prediction accuracy, such as applying robust regression, instead of OLSR.

A limitation of this study is that the MD does not capture local spatial information, such as the shape, size and depth of scotoma, which is important for the sensitivity of detecting progression [29], [30]. In other words, the detectability of progression could be further improved by carrying out point-wise, or cluster wise (superior/inferior hemifield separately) regression, similarly to approaches carried out for measuring 24-2 VF progression [31]–[35].

In conclusion, the proposed method improves the accuracy of progression estimates. This approach can help clinicians to assess and predict patients’ visual function in the parafoveal area.
